# The two sides of chromosomal instability: drivers and brakes in cancer

**DOI:** 10.1038/s41392-024-01767-7

**Published:** 2024-03-29

**Authors:** Rendy Hosea, Sharon Hillary, Sumera Naqvi, Shourong Wu, Vivi Kasim

**Affiliations:** 1 Key Laboratory of Biorheological Science and Technology, Ministry of Education, College of Bioengineering, Chongqing University, Chongqing, 400045 China; 2 The 111 Project Laboratory of Biomechanics and Tissue Repair, College of Bioengineering, Chongqing University, Chongqing, 400044 China; 3 Chongqing Key Laboratory of Translational Research for Cancer Metastasis and Individualized Treatment, Chongqing University Cancer Hospital, Chongqing University, Chongqing, 400030 China

**Keywords:** Cell biology, Cancer, Oncology

## Abstract

Chromosomal instability (CIN) is a hallmark of cancer and is associated with tumor cell malignancy. CIN triggers a chain reaction in cells leading to chromosomal abnormalities, including deviations from the normal chromosome number or structural changes in chromosomes. CIN arises from errors in DNA replication and chromosome segregation during cell division, leading to the formation of cells with abnormal number and/or structure of chromosomes. Errors in DNA replication result from abnormal replication licensing as well as replication stress, such as double-strand breaks and stalled replication forks; meanwhile, errors in chromosome segregation stem from defects in chromosome segregation machinery, including centrosome amplification, erroneous microtubule–kinetochore attachments, spindle assembly checkpoint, or defective sister chromatids cohesion. In normal cells, CIN is deleterious and is associated with DNA damage, proteotoxic stress, metabolic alteration, cell cycle arrest, and senescence. Paradoxically, despite these negative consequences, CIN is one of the hallmarks of cancer found in over 90% of solid tumors and in blood cancers. Furthermore, CIN could endow tumors with enhanced adaptation capabilities due to increased intratumor heterogeneity, thereby facilitating adaptive resistance to therapies; however, excessive CIN could induce tumor cells death, leading to the “just-right” model for CIN in tumors. Elucidating the complex nature of CIN is crucial for understanding the dynamics of tumorigenesis and for developing effective anti-tumor treatments. This review provides an overview of causes and consequences of CIN, as well as the paradox of CIN, a phenomenon that continues to perplex researchers. Finally, this review explores the potential of CIN-based anti-tumor therapy.

## Introduction

Cancer is a widespread and devastating disease, which according to the World Health Organization claimed 10 million lives in 2020.^1^ Cancer is closely associated with mutations and aberrant expressions of a series of oncogenes and tumor suppressor genes. For many years, studies have focused on identifying genes that influence tumorigenesis, such as oncogenes and tumor suppressor genes.^2^ However, it has become increasingly clear that the development and progression of tumors do not rely exclusively on the alteration of a single gene.^3^ Chromosomal instability (CIN), a phenomenon characterized by chromosomal alterations, is observed in over 90% of solid tumors and many blood cancers.^4–6^ These alterations can result in large-scale changes, rearrangements, or disruptions to cellular genetic information, affecting the expression of numerous genes.^2,3,6,7^

The maintenance of genomic stability is a fundamental requirement for the normal functioning of cells.^8–11^ Under normal conditions, cells have developed a series of checkpoints and mechanisms to stringently control the passage of intact and correct genetic information, serving as safeguards that help cells maintain genomic stability and prevent harmful alterations.^8–11^ Thus, CIN, characterized by chromosomal abnormalities, presents a significant challenge to normal cells, often leading to decreased fitness and cell death.^3,12^ Interestingly, in simpler organisms such as bacteria and viruses, while excessive genomic instability is also harmful, a certain increase in genomic instability can be beneficial, as it could increase the heterogeneity of the population, thereby promoting the survival and proliferation of cells with specific genetic aberrations that provide a growth advantage in a stressful environment.^13^ This complex situation reveals that while a moderate level of CIN can be beneficial, extremely high levels result in genetic catastrophe and cell death, highlighting the importance of maintaining a balance.^13^ This delicate balance is not exclusive to simpler organisms but extends to more complex systems, including mammalian cells.^14–20^ Interestingly, a similar paradoxical observation emerges when studying tumor cells, where the role of CIN in tumorigenesis exhibits complexity.^21,22^ Analogous to a double-edged sword, CIN in tumor cells exhibits both tumorigenic and tumor-suppressing effects.^21–26^ On one hand, CIN can promote tumor progression by increasing heterogeneity, thus playing significant roles in tumor development and influencing treatment outcomes,^3,27,28^ while on the other hand, excessive CIN can lead to growth arrest and even cell death.^29^ The precise roles of CIN in tumors remain active areas of research. Elucidating the complex interplay between CIN and tumor progression, as well as treatment response, will not only provide a more comprehensive understanding of a major aspect of tumor development and progression, but also important new perspectives for the development of more effective anti-tumor therapies.

**Fig. 1:**
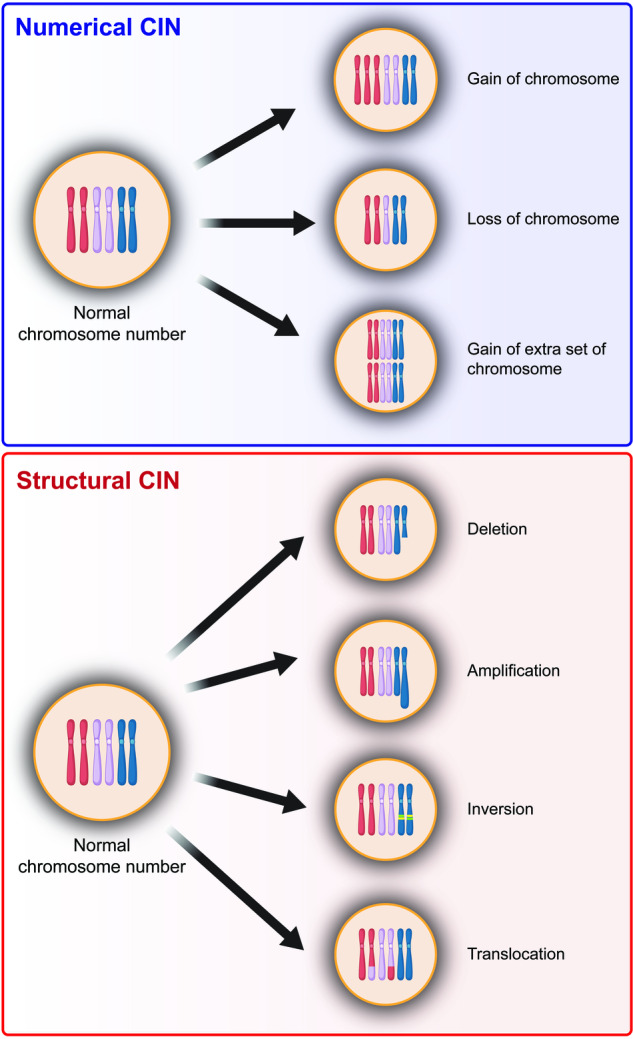
Types of chromosomal instability. CIN is classified into numerical CIN and structural CIN. Numerical CIN corresponds to the gain or loss of whole chromosomes (aneuploidy) or gain of extra set of chromosomes (polyploidy), while structural CIN refers to the gain or loss of chromosome segments due to deletion, amplification, inversion, and translocation

CIN manifests in two distinct forms: numerical CIN and structural CIN (Fig. 1).^30^ Numerical CIN arises from errors in chromosome segregation due to the defects in the mechanisms that guarantee proper sister chromatid segregation, including mitotic checkpoint, centrosome amplification, and abnormalities in microtubules during cell division, and is characterized by the gain or loss of the entire chromosomes.^31^ While numerical CIN does not change the nucleotide sequences within the chromosomes, it alters the copy number of chromosomes, thereby changing the genetic landscape.^32^ In contrast, structural CIN can lead to the gain or loss of chromosomal fragments, which are pieces of chromosomes that have broken off, leading to the alteration in nucleotide sequences of large segments of chromosomes.^32^ This type of CIN is driven by the amplification or deletion of chromosome segments, the formation of extrachromosomal structures, and complex rearrangements of large nucleotide sequences.^31^ Its origins are linked to the mechanisms involved in repairing double-strand breaks (DSBs), managing replication stress, and regulating non-allelic homologous recombination.^31^ Interestingly, structural and numerical CIN often coexist in the majority of tumor cells, thereby creating a complex interplay.^33–35^

Although the study of CIN has a long history in tumor research, recent advancements in next-generation sequencing technology and a deeper understanding of tumor biology have brought CIN back into the spotlight. From a broader biological perspective, genome diversity is a fundamental aspect of evolution and speciation.^36^ It provides the raw material upon which natural selection acts, driving the evolution of new species. In the context of tumors, CIN-induced tumor evolution is crucial for creating this genome diversity. The constant reshuffling of the genome creates a vast pool of genetic variants within the tumor population, known as heterogeneity.^25^ This CIN-induced increased heterogeneity is believed to endow tumors with enhanced evolutionary capabilities due to increased intratumor heterogeneity, facilitating acquisition of malignant phenotypes and adaptive resistance to therapies.^23–26,37–39^ Moreover, accumulating research has revealed other consequences and associations with CIN, such as its links to metastasis and tumor immune regulation.^30^ However, despite these advancements, our understanding of CIN remains incomplete. The complex nature of CIN, its causes and consequences, as well as the paradox of CIN, necessitate a systematic review. This is particularly important, given the potential of CIN as a therapeutic target. Revealing the complex nature of CIN is crucial for understanding one of the major causes of tumor progression, as well as for developing more effective anti-tumor treatments. Therefore, in this review, we aim to provide a comprehensive overview of CIN, exploring its research history, causes, paradoxical nature, and multifaceted influence on tumor biology, as well as discussing the potential and progress of CIN-based anti-tumor therapy.

### Milestones in CIN research

**Fig. 2:**
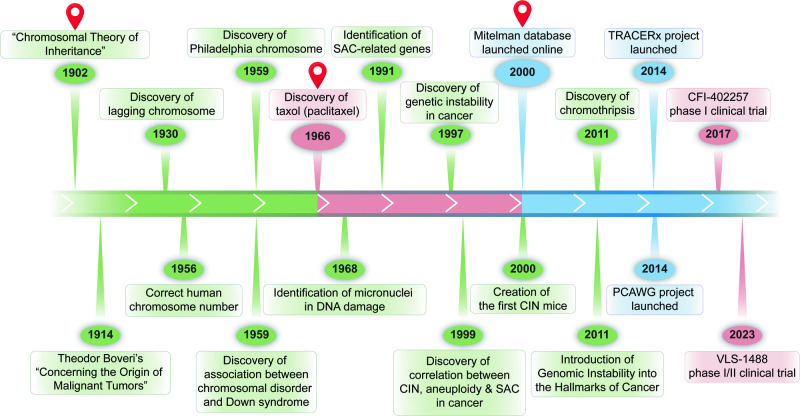
Timeline of key milestones in the CIN research. Green represents milestones in basic CIN research, pink represents milestones in CIN clinical translation, and blue represents milestones in CIN meta-analysis and databases

The study of CIN has evolved over a century, marked by pivotal milestones (Fig. 2). The journey began with Theodor Boveri who, in 1902, performed the first systematic analysis of the effects of aneuploidy on cell and organismal physiology in sea urchins. Boveri observed that embryos resulting from eggs fertilized by two sperms exhibited developmental defects and died, concluding that chromosome abnormality leads to defect in development and lethality, marking the first hypothesis that connects between chromosomal abnormality and disease. His subsequent work in 1914, “Concerning the Origin of Malignant Tumors,” linked chromosomal abnormality to cancer, marking the first hypothesis that connected chromosomal abnormality to cancer.^40,41^ Sixteen years later, Barbara McClintock introduced the terms ‘laggards’ or ‘lagging chromosomes’ to signify chromatin lagging between daughter nuclear masses during anaphase, providing a deeper understanding of chromosomal behavior during cell division.^42^ The field of clinical cytogenetics was initiated in 1956 when Tjio and Levan discovered that humans have 46 chromosomes. This discovery not only corrected the previously held belief of 48 chromosomes, but also paved the way for the study of chromosomal abnormalities in humans.^43^ In 1994, Rieder and colleagues performed their classic experiment using laser ablation, which revealed the role of unattached kinetochores in extending the duration of mitosis, providing crucial insights into the mechanisms of mitotic checkpoint control.^44^

The relation between CIN and diseases was first revealed in 1959, when two significant discoveries were made. Peter Nowell identified the Philadelphia chromosome, a consequence of an abnormal rearrangement between human chromosome 9 and chromosome 22 that could be found in approximately 90% of chronic myeloid leukemia patients, providing the initial evidence of structural chromosome aberration as a malignant factor.^45,46^ In the same year, Lejeune et al. discovered that an additional copy of chromosome 21, a condition now known as trisomy 21, caused Down syndrome.^47^ Lejeune et al. continued his research and identified another chromosomal disorder known as cri du chat (cry of the cat) syndrome, a condition that arises when a segment of chromosome 5 is missing.^48^ These discoveries underscored the detrimental effects associated with chromosome aberrations and emphasized the importance of chromosomal stability.^45,47^ In 1997, Lengauer et al. quantified CIN in human cancer cell lines, proposing its universality across cancers;^49,50^ while Angelika Amon’s works from 1999 until 2010 elucidated the molecular aspects of checkpoint crucial in CIN.^51,52^ These works provided valuable insights regarding the widespread nature of CIN and its molecular mechanisms in cancer, thereby underscoring the significance of CIN in cancer biology. This eventually leads to the recognition of CIN as one of the hallmarks of cancer in 2011.^49^ Meanwhile, in 2018, Bakhoum et al. provided compelling evidence that CIN could drive tumor metastasis, significantly advancing our understanding of the role of CIN in tumor progression.^53^

The beginning of the 21st century marked another crucial milestone in CIN research. In 2000, Max Dobles et al. developed the first CIN mice model, enabling in vivo studies of CIN.^54^ In the same year, Felix Mitelman launched The Mitelman Database of Chromosome Aberrations and Gene Fusions in Cancer, which provides a valuable resource for CIN research, marking the opening of the era of meta-analysis in CIN studies.^55^ This was continued with the initiation of Pan-Cancer Analysis of Whole Genomes (PCAWG) by the International Cancer Genome Consortium (ICGC) in 2014.^31,33,56,57^ Concurrently, the TRAcking Cancer Evolution through therapy/Rx (TRACERx) clinical study was launched under the guidance of Charles Swanton in 2014. These initiatives marked a significant shift towards large-scale, collaborative efforts in understanding CIN, and paved the way for the development of therapeutic interventions targeting CIN.^6,58–62^ The discovery of Taxol in 1966 marked a significant milestone in anti-tumor treatment, setting the stage for a novel anti-tumor therapeutic strategy. It was the first drug to successfully demonstrate the potential of targeting mitosis, laying the groundwork for treatments based on CIN.^63^ Then, half a century later in 2017, this strategy was further realized with the first clinical trial for drugs targeting CIN, starting with the phase I trial of CFI-402257. Although this drug has not yet received full approval, it was granted Fast Track Designation by the FDA in 2023.^64–67^ To date, two anti-tumor therapies targeting CIN has been approved, while more than 50 are in clinical trial Phase I/II, with the most recent one initiated in October 2023 for VLS-1488.^68^ These developments have significantly advanced the translation of CIN research into potential therapeutic interventions. To date, research into CIN continues to progress, with each new discovery providing further insight into this complex field and opening up new avenues for potential cancer treatments.

### Causes of CIN

**Fig. 3:**
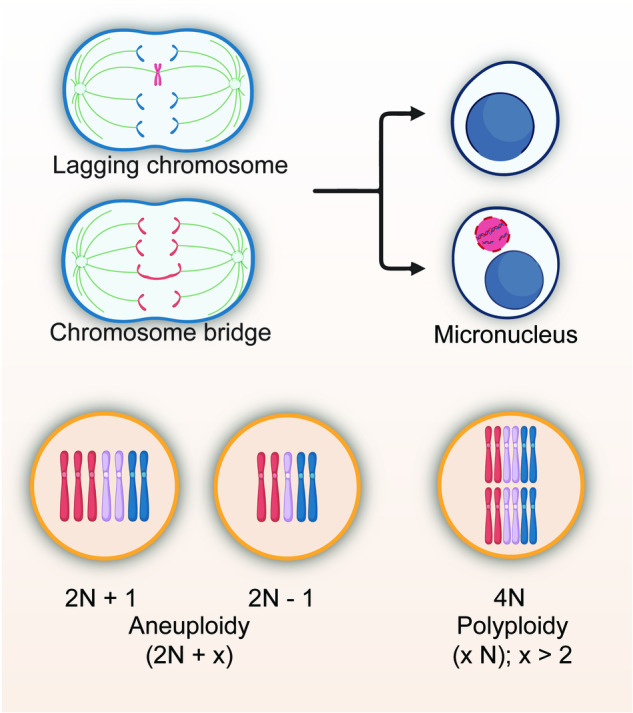
Indicators of CIN. Example of indicators commonly used to assess CIN, including lagging chromosomes, chromosome bridges, micronuclei, aneuploidy, and polyploidy

CIN is characterized by changes in chromosome structure and number during cell division.^30,32,69^ There are several key indicators of CIN, including lagging chromosomes, chromosome bridges, micronuclei, aneuploidy, and polyploidy.^70–86^ As will be discussed below, aberrant spindle assembly checkpoint (SAC) activity, impaired sister chromatid segregation, aberrant centrosome number, and microtubule-kinetochore attachment error could lead to chromosome missegregation.^87–112^ This could in turn increase the formation of lagging chromosomes, which are chromosome that moves to the poles of the cell during cell division slower than other chromosomes, and chromosome bridges, which are structures formed when part of sister chromatids intertwines and fails to completely segregate.^113,114^ Lagging chromosomes and chromosome bridges subsequently could lead to the formation of micronucleus, a small, extra-nuclear body that contains chromosomal fragments or whole chromosome that are not incorporated into the main nucleus.^71,115–117^ Furthermore, chromosome missegregation, along with replication stress, sister chromatid defect, and abnormal centrosome number, could also lead to numerical CIN, as they could promote the occurrence of aneuploidy, a condition where a cell has an abnormal number of individual chromosomes, as well as polyploidy, a condition in where a cell has multiple sets of chromosomes.^34,113,114,118,119^ These indicators reflect the level of CIN, and are commonly used to assess and study CIN in a cell population (Fig. 3).

**Fig. 4:**
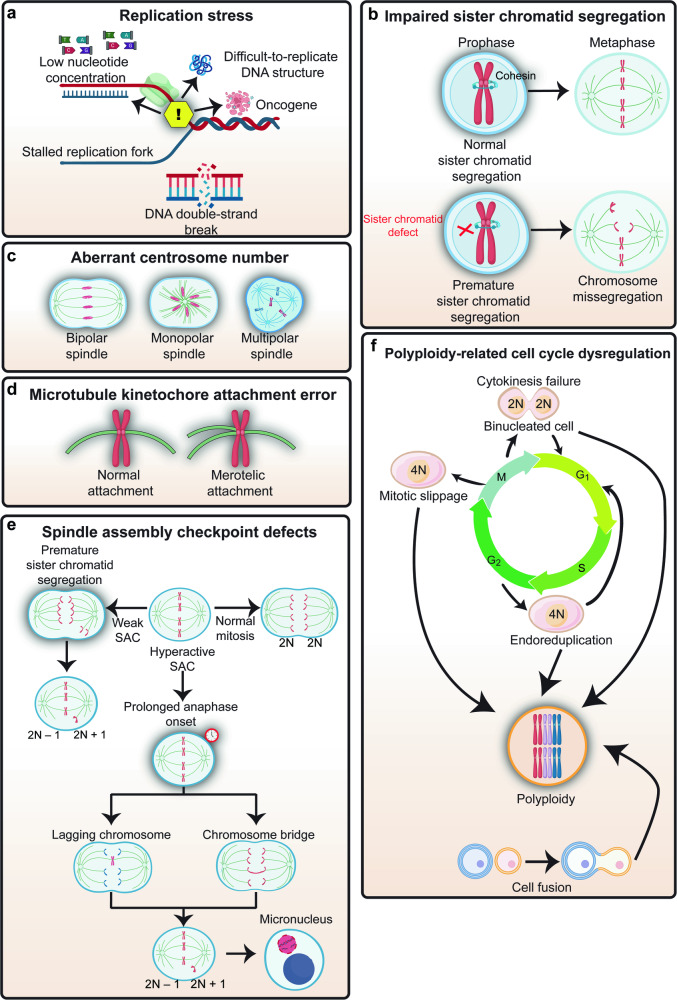
Causes of CIN. **a** Replication stress leads to stalling and collapse of replication forks, which results in DSBs. **b** Sister chromatid defect allows premature separation of sister chromatids before full alignment, leading to chromosome missegregation. **c** Aberrant centrosome number such as monopolar spindle and multipolar spindle could lead to aneuploidy. **d** Microtubule kinetochore attachment error causes failure to form bi-orientation, where each kinetochore is attached to microtubules from only one spindle pole, leading to chromosome missegregation. **e** Aberrant SAC could lead to aneuploidy, as weakened SAC causes premature chromatid separation, while hyperactivated SAC results in a lagging chromosome. **f** Extra set of chromosomes as seen in polyploidy could arise from cytokinesis failure, mitotic slippage, or endoreduplication

The causes of CIN are multifaceted and can be attributed to a variety of factors (Fig. 4). At its core, CIN is often the result of errors in DNA replication, which can lead to the formation of cells with incomplete or excess genetic material; as well as errors in chromosome segregation during cell division, which can lead to the formation of cells with an abnormal number of chromosomes.^120,121^ Errors in DNA replication can arise from the abnormal replication licensing as well as replication stress, such as DSBs and stalled replication forks; while errors in chromosome segregation can arise from defects in chromosome segregation machinery, including issues with centrosome amplification, erroneous microtubule-kinetochore attachments, and defects affecting the mitotic checkpoint or impaired sister chromatid segregation. Furthermore, some events induced by CIN could further trigger instability of chromosomes. For instance, chromothripsis is an event of incorporation of chromosome fragments originated from micronuclear chromosome into nuclear chromosome, causing rearrangement of nuclear chromosome.^78,115,122–133^ Thus, while chromothripsis itself is a consequence of CIN, it could also be a cause of subsequent CIN.

#### Replication stress and defective DNA repair

Replication stress is a condition that occurs when the DNA replication machinery is disrupted during the S phase of the cell cycle, leading to a stalled replication fork.^134,135^ Replication stress can be driven by oncogenes, low nucleotide concentrations, and DNA sequences or structures that are difficult to replicate.^136^ In response to replication stress, cells activate the DNA damage response (DDR), a cellular response that requires a network of repair proteins. This network includes key proteins such as ataxia telangiectasia mutated (ATM) and ataxia telangiectasia and Rad3 related (ATR), which are kinases that help the stabilization of the stalled replication fork, preventing it from collapsing.^137,138^ As has been observed in various precancerous and cancerous lesions, failure in resolving the stalled replication forks can cause DSBs, and subsequently, rearrangements of parts of chromosomes (translocations) or deletions, thus contributing to structural CIN.^34,137–144^

In addition to the DDR pathway, other DNA repair-related factors, such as Fanconi anemia (FA) proteins are also important for preventing CIN.^145–147^ The FA pathway is important for repairing interstrand cross-links, which are toxic lesions that prevent DNA strand separation, block replication, and hinder transcription, thereby playing a critical role in responding to replication stress and maintaining chromosome stability.^148–150^ Defects in the FA pathway, specifically in the *Fanconi anemia complementation group D2* (FANCD2), can lead to increased translocations and abnormal chromatin structures, contributing to structural CIN.^42,147^ Moreover, replication stress can also lead to lagging chromosomes and micronuclei.^34,118,151,152^ Together, these studies demonstrated that replication stress and defects in DNA repair systems contribute to the generation of structural and numerical CIN.

#### Impaired sister chromatid segregation

The separation of the chromosomes at anaphase requires the loss of sister cohesion in a timely manner. This is facilitated by the cohesin complex, a multi-protein complex composed of four core subunits: either stromal antigen 1 (STAG1) or stromal antigen 2 (STAG2), structural maintenance of chromosomes 1A (SMC1A), structural maintenance of chromosomes 3 (SMC3), and RAD21 cohesin complex component (RAD21).^153–158^ During prophase, the bulk of the cohesin complex, which consists of a ring-shaped structure formed by SMC1A, SMC3, RAD21, and STAG1 in the chromosome arm is removed.^159,160^ This process involves several proteins, including the WAPL cohesin release factor (WAPL), PDS5 cohesin-associated factor (PDS5), and polo-like kinase 1 (PLK1). These proteins assist in opening the cohesin ring, facilitating its removal from the chromosome arms.^161^ At anaphase, when separase is activated, the cohesin complex consisting of SMC1A, SMC3, RAD21, and STAG2 in the centromere was cleaved at the RAD21 subunit, leading to the opening of the cohesin ring at the centromere and the final separation of sister chromatids.^153,159^

Genetic alterations of any of the cohesin subunits, including mutations and inactivation, have been associated with CIN in various human tumors, as this dysregulation in turn leads to aberrant chromatid cohesion and allows premature separation of sister chromatids before full alignment, leading to chromosome bridge, lagging chromosome, and micronuclei.^153^ Defects in cohesin subunits, such as *STAG1* or *SMC1A*, cause premature separation of chromosome arms and increased aneuploidy.^87,88^ Moreover, *WAPL* overexpression induces premature separation of chromosome arms, thereby increasing the rate of chromosome bridge and micronuclei;^89^ while *PDS5* defect disrupts the regulation of cohesin ring removal from chromosome arms, leading to increased DSBs through an as-yet-unknown mechanism.^90^

Meanwhile, mutations in *STAG2* as well as *RAD21*, a core component of cohesin complex at the centromere crucial for holding the centromeres of sister chromatids together from the time of DNA replication in S phase until their segregation in mitosis, also cause premature separation of sister chromatids and subsequently increase aneuploidy.^91–93^ Interestingly, mutation in *STAG2* could also disrupt the interaction between cohesin and the replication machinery, thereby triggering DSBs and subsequently translocation by increasing stalling and collapse of replication forks.^94,95^ Furthermore, mutation in *RAD21*, a core component of the cohesin complex that plays a crucial role in holding the centromeres of sister chromatids together from the time of DNA replication in S phase to their segregation in mitosis, induces premature separation of sister chromatids, and increased aneuploid.^92^ Together, these studies demonstrated that sister chromatid defect contributes to the generation of structural and numerical CIN.

#### Aberrant centrosome number

CIN can also arise from aberrant centrosome amplification and separation, both of which are critical processes in cell division. Failure in centrosome amplification, for example due to the defects in specific motor proteins such as kinesin family member 2A (KIF2A), kinesin-like protein at 10A (KLP10A), and kinesin-like protein at 67A (KLP67A), or defects in centrosome proteins such as γ-tubulin, gamma complex component 2 (GCP2), and gamma complex component 3 (GCP3), can cause formation of monopolar spindle due to poor centriole separation.^162^ Monopolar spindle in turn leads to improper sister chromatid separation, as there is only one pole for them to move towards.^96^ Eventually, all chromosomes end up in a single daughter cell when the cell completely divides, resulting in the formation of polyploid cell.^163^

Improper timing of centrosome separation prior to cell division, both delayed and accelerated centrosome separation, would also lead to the formation of monopolar spindle.^164,165^ Loss of *ubiquitin-specific peptidase 44* (*USP44*), a deubiquitinase that localizes at the centrosome, results in incomplete centrosome separation as well as increased monopolar spindle, lagging chromosome, and chromosome bridge. Moreover, *USP44* knockout mice are prone to increase in numerical CIN as observed by elevated levels of aneuploidy.^166^ Defective centrosome separation can also occur in cells with overexpressed *kinesin family member 11* (*KIF11*), a motor protein that drives centrosome separation. *KIF11* overexpression can disrupt the normal timing and coordination of centrosome separation, by causing centrosomes to separate too quickly before the completion of centrosome duplication. In such cases, the spindle poles would be formed by imperfectly duplicated centrosomes, leading to the formation of monopolar spindle, and eventually, polyploidy.^97^

Meanwhile, the mitogen-activated protein kinase (MAPK) pathway, known for its role in cell proliferation, differentiation, and survival, has been implicated in the induction of CIN through centrosome amplification, as constitutive activation of MAPK through *rat sarcoma virus* (*ras*)-overexpression resulted in the increase of centrosome amplification, leading to multipolar spindle.^167^ Furthermore, overexpression of *polo-like kinase 4* (*PLK4*), a master regulator of centrosome amplification, can result in overduplication of centrosome.^98^ This consequently lead to the formation of cell with multipolar spindles. When anaphase occurs in these cells, the chromosomes are separated abnormally, resulting in aneuploid daughter cells.^98^ Together, defects in centrosome duplication and separation contribute to the formation of monopolar and multipolar spindles, which are frequently observed in human tumors and are associated with CIN.^168–173^

#### Microtubule kinetochore attachment error

The process of spindle microtubules binding to kinetochores is asynchronous and stochastic, occurring at different times and in a random manner. This randomness and lack of synchronization can sometimes lead to erroneous attachments, such as merotelic attachments where a single kinetochore binds to microtubules anchored at both spindle poles, and could lead to chromosome missegregation.^113,174–177^ Despite the erroneous nature of merotelic attachments, cells often manage to segregate these chromosomes correctly during anaphase. This is due to the cell mechanism that corrects these erroneous kinetochore-microtubule (K-MT) attachments by converting them into bi-oriented attachments, where each kinetochore is attached to microtubules from only one spindle pole.^178–180^

The efficient correction of merotelic attachments requires the dynamic turnover of K-MT interactions. The optimal stability of K-MT attachments, which is neither too loose nor too hyperstable, is crucial for this process. A decrease in this turnover rate could result in persistent merotelic attachments and increased chromosome segregation errors, such as lagging chromosomes.^99,100^ For instance, some tumors with CIN are characterized by hyperstable K-MT interactions, a state that is more stable compared to chromosomally euploid cells, leading to failure in correcting merotelic attachments and increased CIN; while reducing the K-MT attachment stability from this hyperstable state can restore normal chromosome segregation in cells with CIN.^99,100,181^ However, while previous studies have shown that loss of *STAG2*, a cohesin subunit that has been reported to have roles beyond sister chromatids cohesion, results in hyperstabilized K-MT attachments, the exact molecular mechanisms are not fully understood.^182,183^ To fully characterize the contribution of microtubule-kinetochore attachment errors in CIN, a more detailed study of the complex and dynamic process of spindle microtubules binding to kinetochores is required.

#### SAC defects

The primary goal of a cell undergoing mitosis is to segregate the replicated chromosomes into two new daughter cells. This is achieved through the attachment of chromosomes to microtubules of the mitotic spindle apparatus.^184^ Chromosomes attach to the ends of microtubules at kinetochores, which are specialized protein structures that bind to the chromatin centromere.^184^ Normally, each chromosome has two kinetochores, and it is essential for mitotic cells to form bi-orientation.^184^ This state is achieved when each sister kinetochore binds microtubules oriented toward opposite spindle poles.

A checkpoint mechanism known as the SAC delays the separation of the sister chromatids at anaphase until every kinetochore has correctly attached to spindle microtubules and all sister chromatids have aligned at the metaphase equatorial plate.^185–188^ Thus, the SAC is a safeguard for guaranteeing chromosome bi-orientation on the mitotic spindle by monitoring the proper kinetochore attachment as well as chromosome alignment. As long as improperly attached or unaligned chromosomes remain, SAC halts cells in mitosis and prevents their progress into the final phases of cell division.^185–188^ Components of the SAC, including mitotic arrest deficient 2 (MAD2),^107,189–192^ budding uninhibited by benzimidazoles 1 (BUB1),^193–196^ budding uninhibited by benzimidazoles 1 beta (BUBR1),^110,195,197^ and budding uninhibited by benzimidazoles 3 (BUB3),^193,194,198–202^ migrate to unattached kinetochores and form mitotic checkpoint complex (MCC) along with cell division cycle 20 (CDC20). MCC is a key effector of SAC that inhibits the activation of the CDC20-bound anaphase-promoting complex/cyclosome (APC/C^Cdc20^), an E3 ubiquitin ligase that targets cyclin B and securin for degradation by the proteasome.^185–187,203–211^ Once sister chromatids have properly attached and aligned, the SAC is inactivated, allowing the MCC to dissociate, freeing CDC20 to activate the APC/C.^212^ The activation of the APC/C^CDC20^ triggers securin and cyclin B degradation.^203,207,209,213^ Securin destruction frees separase, an enzyme that cleaves and inactivates the cohesin complex, allowing sister chromatid separation and the onset of anaphase.^203^ Meanwhile, cyclin B degradation inactivates cyclin-dependent kinase 1 (Cdk1), allowing the cells to proceed to mitotic exit and complete cell division.^185,186,214,215^

In eukaryotic cells, SAC plays a crucial role in genomic integrity and its abnormality leads to chromosome segregation errors.^10,101^ Defects in SAC result in the failure of proper monitoring and controlling the timing of sister chromatid segregation.^10,101,102^ This in turn leads to increased chromosomal abnormalities, such as chromosome bridge and lagging chromosome, and eventually, errors in equal distribution of genetic material to daughter cells.^103–105^ Moreover, as described above, lagging chromosome, as well as chromosome bridge, could reassemble and form micronucleus, a nucleus-like structure consisting of a bilayer membrane covering a piece of extrachromosomal DNA.^175^

Cells lacking MAD2, an SAC component, can proliferate in vitro and in vivo but with increased levels of CIN.^103,104^ Moreover, weakening the checkpoint in mice by partially reducing the expression of various SAC genes including MAD1, MAD2, BUB1, BUBR1, and BUB3, results in premature separation of sister chromatids, chromosome missegregation, and subsequently, CIN.^106–112^ In addition to the SAC components, the CDK pathway also plays a significant role in CIN. Gao et al. reported that CUE domain containing 2 (CUEDC2) is phosphorylated by CDK1 during mitosis. This phosphorylated CUEDC2 promotes spindle checkpoint inactivation by promoting MCC dissociation from the APC/C, leading to premature inactivation of SAC and increased CIN.^216^ Furthermore, chromosome missegregation can be caused by mutations that weaken the SAC, which subsequently results in premature anaphase onset.^105^ However, mutations in SAC genes are rarely found in human tumors, suggesting that while SAC mutation is one of contributors to CIN in tumor cells, aberrant transcriptional, post-translational modification, and epigenetic regulations might also contribute to SAC defects.^121,217–219^

Interestingly, while weakened SAC can cause CIN, overactivity of the checkpoint induced by, for example, overexpression of SAC gene such as *MAD2*, or knockdown of genes involved in SAC silencing pathways such as *p31/comet* or *TRIP3*, can also induce CIN.^220–224^ Similar to SAC defect, SAC hyperactivation could lead to the increase of chromosome bridge, lagging chromosome, and micronucleus.^220–225^ However, in contrast to weakened SAC which accelerates mitotic progression and tumor cells proliferation, SAC hyperactivation delays the onset of anaphase and prolongs mitotic arrest.^220–225^ Furthermore, unlike chromosome missegregation induced by SAC defect, which stems from the premature anaphase progression before the erroneous chromosomes-microtubules attachments are corrected, the mechanism of SAC hyperactivation-induced chromosome missegregation is not entirely understood. One possible explanation is that persistent SAC signaling could lead to cohesion fatigue, where the cohesin complexes that hold sister chromatids together become exhausted over time, resulting in aberrant sister chromatid segregation.^226,227^ Together, while the SAC plays a vital role in ensuring accurate chromosome segregation during mitosis, both its defect and hyperactivation can paradoxically lead to CIN.

#### Polyploidy-related cell cycle dysregulation

Polyploidy is a condition where a cell has multiple sets of chromosomes and could be both a consequence as well as a cause of CIN.^228–232^ Polyploidy can occur due to various reasons, including cytokinesis failure, mitotic slippage, endoreduplication, or cell fusion.^233^ Cytokinesis failure occurs when daughter cells fail to separate after accomplishing telophase.^74,234–236^ This can happen due to various reasons, such as problems with the contractile ring that separates the two daughter cells or the presence of chromosome bridges that physically prevent the cells from separating.^234^ When cytokinesis failure occurs, the two daughter cells remain connected and form a binucleated cell with twice of the normal number of chromosomes.^234^ Furthermore, polyploidy can be caused by mitotic slippage, which is a process of premature mitotic exit. This can occur when the SAC activity is weakened, leading to the misinterpretation that all chromosomes are correctly attached to the spindle and the failure to inhibit the activation of the APC/C^CDC20^.^108,237,238^ This failure then promotes the premature degradation of cyclin B1, which in turn leads to a decrease in Cdk1 activity, and, as a consequence, promotes the onset of anaphase and premature exit from mitosis without proper chromosome segregation.^108,237,238^

Endoreduplication is a process in which cells undergo multiple rounds of DNA replication without mitosis, resulting in cells with multiple copies of their genome.^239^ Endoreduplication can occur due to various reasons, such as problems with the cell cycle machinery.^239^ One key cell cycle machinery associated with this endoreduplication is a defect in the pre-replication complex (pre-RC).^240^ The pre-RC, which includes the origin recognition complex, cell division cycle 6, chromatin licensing and DNA replication factor 1 (CDT1), and minichromosome maintenance complex 2-7, assembles at replication origins during G1 phase to license DNA replication at S phase.^240^ This complex then dissociates from the replication origins after DNA replication is started to prevent another round of DNA replication before the cell completes cytokinesis, thereby guaranteeing the proper number of chromosomes being passed to daughter cells.^240^ Hence, dysregulation in pre-RC could lead to whole-genome doubling (WGD), a form of polyploidy.^228,241–246^

Cell fusion is a process in which two or more cells fuse together to form a single cell with multiple nuclei, also known as a synkaryote.^239,247^ This can occur due to various reasons, such as exposure to certain viruses or chemicals.^239,247^ Following fusion, the parental chromosomes mix and redistribute to the fused cells, thereby producing polyploid fused cells.^239,247^ Therefore, cell fusion can also result in polyploid cells and contribute to CIN.

The increase in polyploidy, therefore, signifies an increase in CIN, underlining the critical role these cellular processes play in causing CIN. However, as mentioned above, besides as a consequence of CIN, polyploidy is also an important cause of CIN. The extra set of chromosomes in a cell can lead to errors in chromosome segregation during subsequent cell division, forming aneuploid cells with abnormal chromosome numbers.^74,248–251^ For instance, a study using tetraploid cells demonstrated that these cells, which contain twice the number of chromosome sets along with two extra centrosomes, can lead to the formation of multipolar spindles. This, in turn, results in the formation of aneuploid cells.^252^ On the other hand, cells with CIN can also become polyploid due to errors during cell cycle dysregulation as mentioned above.^253^ Together, this highlights the complex interplay between polyploidy and CIN.

### CIN paradox

Maintaining genomic stability is essential for the normal functioning of the cells, and for ensuring the accurate transmission of genetic information to progeny, thereby preserving the continuity of the species.^8–11^ Normal cells have intrinsic potentials for maintaining their genome integrity through various mechanisms, such as the ability to repair their damaged DNA, as well as for preventing the passage of damaged, unrepairable DNA to their progenies by triggering apoptosis.^254^ Given the critical role of genomic stability, any deviation from this state can have deleterious consequences. One such challenge is CIN, which can be detrimental when present in normal cells, as it could decrease cellular fitness.^3,12^ As observed by Theodor Boveri over a century ago, chromosomal abnormalities are typically intolerable in normal cells, often culminating in cell death.^41^ However, it is essential to note that CIN is not universally detrimental. There are examples, notably in simpler organisms like bacteria, viruses, and fungi where elevated genomic instability can confer advantages in stressful environments.^13^

For instance, clinical isolates of the yeast *Candida albicans* that are resistant to the anti-fungal drug fluconazole carry extra copies of chromosome 5, where genes encoding the drug target, *lanosterol 14-α-demethylase* (*ERG11*), and a main regulator of drug efflux pumps, *transcription activator of CDR genes 1* (*TAC1*), are located.^255^ This indicates that CIN could provide drug resistance through the increased expression of these genes.^256^ Furthermore, in other yeasts, *Saccharomyces cerevisiae* and *Candida glabrata*, increased CIN-induced aneuploidy results in phenotypic advantages by promoting their resistance to fluconazole.^257–259^ Similarly, in bacteria and viruses, elevated genomic instability benefits the population in stressful environments by promoting the survival and proliferation of cells with specific genetic aberrations that confer a growth advantage.^13^ This suggests a nuanced perspective on CIN, highlighting its potential benefits under specific circumstances. However, it is important to emphasize that the intensity of CIN cannot exceed certain thresholds. Indeed, in bacteria and viruses, cells with drastic instability never become dominant in a population, as their excessive instability levels exceed the cellular threshold, leading to genetic catastrophe and cell death.^13^ This observation suggests that while moderate CIN can be beneficial, excessive CIN may lead to genetic catastrophe and is lethal. Therefore, understanding the balance between beneficial and detrimental effects of CIN is crucial to comprehend its role, not only in simpler organisms but also in more complex systems like mammalian cells.^14–20^

With the advancement of our knowledge regarding tumor biology, a similar observation emerges, where CIN resembles a double-edged sword (Table 1). On one hand, CIN can promote tumorigenesis. As exemplified in yeast and mammalian cells above, CIN could contribute to clonal evolution, providing selective advantages under stressful conditions encountered by tumor cells.^257,260,261^ This clonal evolution, driven by CIN, can be a key factor in promoting tumorigenesis, as it not only helps tumor cells to survive in harsh environments but can also foster the evolution of the clones with the most tumorigenic phenotypes, that is, clones that have new karyotype that brings them growth advantage and the ability to outcompete others.^260^ This is also supported by studies using animal models. For example, mice carrying heterozygous deletions of SAC genes, such as *MAD1*, *MAD2*, and *BUB1B*, exhibit increased CIN and develop spontaneous tumors.^106–108^ Similar evidence comes from human patients with mosaic variegated aneuploidy syndrome (MVA), which is characterized by increased CIN and a predisposition to childhood cancer.^109,110,262,263^ These results suggest that tumor cells may exploit CIN to harness the potential of clonal evolution for optimal adaptation. However, on the other hand, CIN has also been reported to have anti-proliferative effects,^264–266^ and can induce cell death,^267,268^ senescence,^269–271^ as well as anti-tumor immune response.^270,272,273^ Clinical observations further complicate the picture, with high CIN signatures in various tumors associated with improved prognosis.^274–279^ These observations have led to the establishment of the “just-right” hypothesis, proposing a moderate level of CIN that benefits tumorigenesis and tumor progression. However, while the concept of how populations with genetic instability evolve over time has been observed in a study based on mathematical modeling,^280,281^ experimental evidence to support the “just-right” hypothesis for the relation between CIN level and tumor cell fate determination, as well as the molecular mechanisms underlying it are still lacking. One possible explanation for this, at least in part, is the use of different models to elucidate the role of CIN in cancers, emphasizing the need for further research. In addition, it is noteworthy that the specific context of aneuploidy induced by CIN can also influence tumor cell fate. While tumor cells may be able to tolerate the effects of additional chromosomes, excessive loss of chromosomes that contain essential genes for cell survival is detrimental. Therefore, the balance between gain and loss of chromosomes is crucial for tumor cell viability.^282,283^

**Table 1:** Roles of CIN in cancer

Phenotype	Model	CIN indicators	Mechanism	Ref
Tumor-promoting	*AAA-Cdc20* heterozygous MEFs	Increased aneuploidy	Weakened SAC function	^456^
Tumor-promoting	*Apc*^*Min*/+^*BubR1*^+/–^mice	Increased aneuploidy, polyploidy, sister chromatid premature separation	Weakened SAC function	^457,458^
Tumor-promoting	*AURKA*-overexpressed mice	Increased aneuploidy, polyploidy, chromosome missegregation, sister chromatid premature separation	Centrosome amplification	^459–462^
Tumor-promoting	*BUB1* haploinsufficient mice	Increased aneuploidy and chromosome missegregation	Weakened SAC function	^463^
Tumor-promoting	*BUB3* haploinsufficient MEFs	Increased aneuploidy, sister chromatid premature separation	Weakened SAC function	^199^
Tumor-promoting	*BUBR1* haploinsufficient mice	Increased polyploidy	Weakened SAC function	^464,465^
Tumor-promoting	*Cyclin B-*overexpressed MEFs	Increased aneuploidy, chromosome missegregation	Prolonged mitotic exit	^96^
Tumor-promoting	*CENP-E* heterozygous MEFs	Increased aneuploidy, polyploidy, chromosome missegregation	Microtubule-kinetochore attachment error	^466^
Tumor-promoting	*HEC1*-overexpressed mice	Increased aneuploidy, polyploidy, chromosome breaks	SAC hyperactivation	^467^
Tumor-promoting	*MAD1* heterozygous deletion mice	Increased aneuploidy	Weakened SAC function	^106^
Tumor-promoting	*MAD2* haploinsufficient cells	Increased aneuploidy, sister chromatid premature separation, chromosome missegregation	Weakened SAC function	^107^
Tumor-promoting	*Securin* homozygous deletion mice	Increased aneuploidy, polyploidy, sister chromatid premature separation	Premature sister chromatid separation	^468,469^
Tumor-promoting	*TPX2* heterozygous mice	Increased aneuploidy, chromosome missegregation,	Disrupted normal microtubule polymerization	^470^
Tumor-promoting	*UbcH10*-overexpressed mice	Increased aneuploidy, chromosome missegregation, centrosome amplification	Premature sister chromatid separation	^471,472^
Tumor-suppressing	*CDH1* heterozygous mice	Increased replication stress, aneuploidy, polyploidy,	Prolonged mitosis	^473^
Tumor-suppressing	*STAG1* heterozygous deletion mice	Increased aneuploidy, polyploidy, chromosome missegregation	Impaired sister chromatid segregation	^474^
Tumor-suppressing	*MAD2-*overexpressed MEFs	Increased aneuploidy, polyploidy, chromosomal breaks	SAC hyperactivation	^220^
Tumor-suppressing	*α-GSU PTTG*-overexpressed mice	Increased aneuploidy, polyploidy, sister chromatid premature separation	Prolonged mitosis	^475,476^
Tumor-suppressing	*PLK1* overexpressed mice	Increased polyploidy, lagging chromosome, sister chromatid premature separation, micronucleus, cytokinesis failure	SAC hyperactivation	^477^
Tumor-suppressing	*MAD2* overexpression	Increased lagging chromosome, micronucleus, polyploidy	SAC hyperactivation	^220^
Tumor-suppressing	Ionizing radiation-exposed cells	Increased lagging chromosome, micronucleus	DNA DSBs	^277^
Tumor-suppressing	Paclitaxel-treated cells	Increased multipolar spindles, lagging chromosome	Prolonged mitosis	^409^

**Fig. 5:**
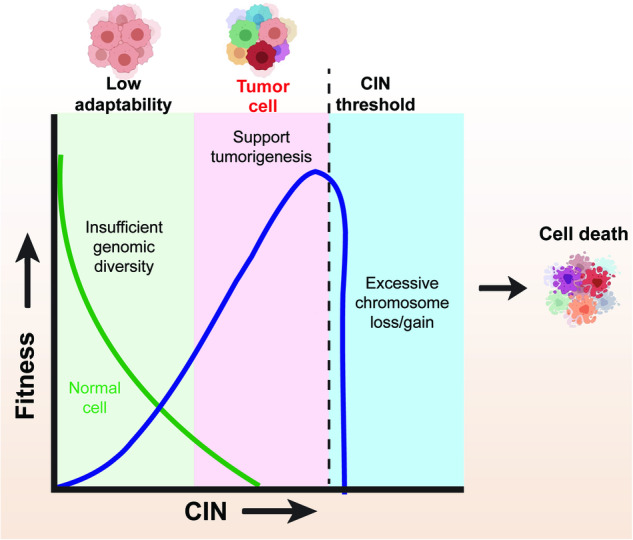
CIN paradox according to the “just-right” model. A moderate, “just-right” level” for CIN could induce a tumor-promoting phenotype by increasing tumor cells adaptability, while excessive CIN is deleterious to tumor cells due to excessive chromosome gain or loss leading to cell death

Together, the role of CIN in tumorigenesis is complex and paradoxical (Fig. 5), influenced by factors such as the degree of CIN and the specific conditions faced by tumor cells. The intricate balance between the harmful and advantageous effects of CIN underscores the complexity of CIN paradox, emphasizes the need for further research. In the following section, we will discuss further the consequences of CIN and how its seemingly negative effects can, under certain circumstances, confer advantages to tumor cells.

### The multifaceted impacts of CIN on tumor biology

**Fig. 6:**
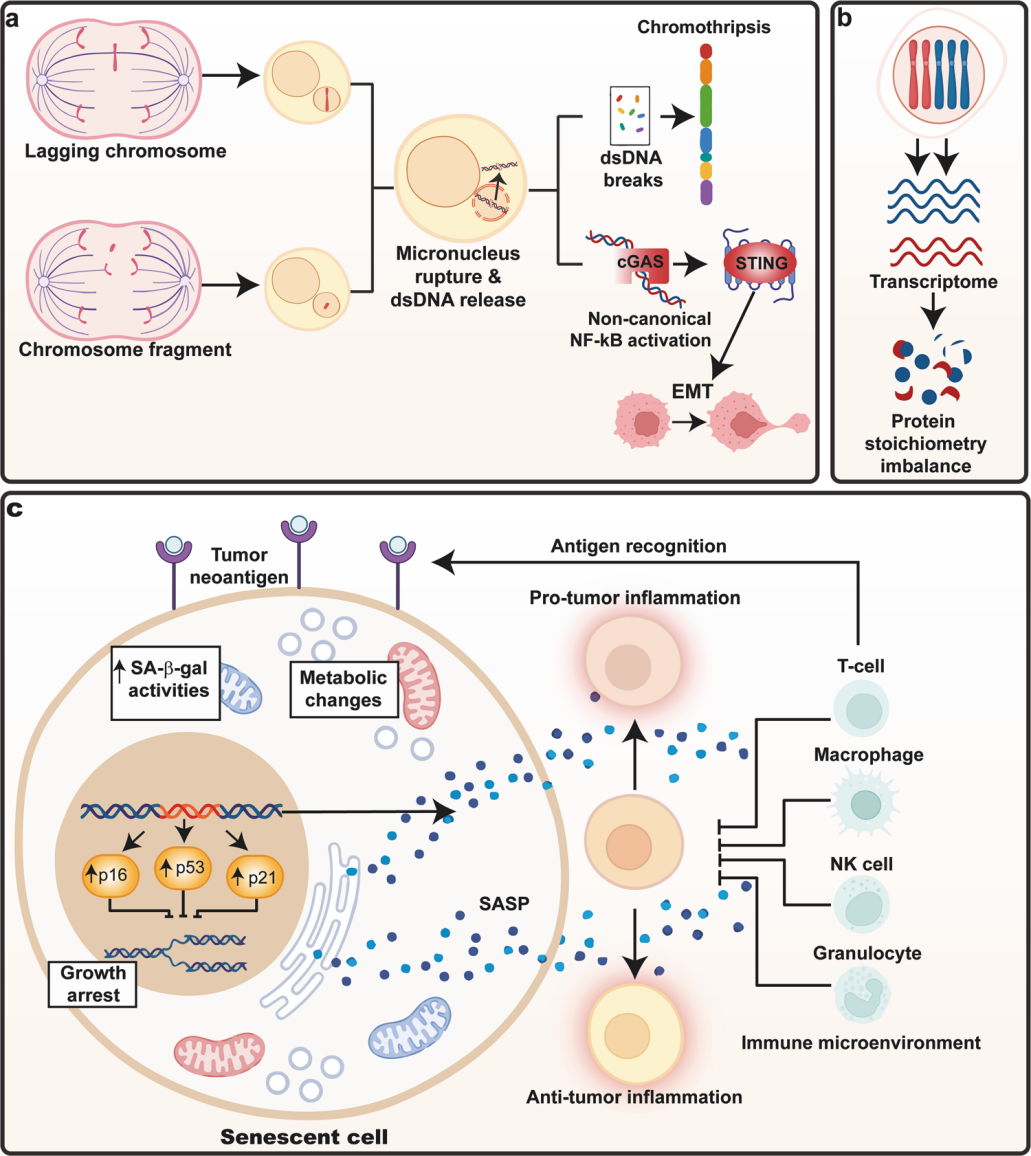
Consequences of CIN. **a** Chromothripsis, also known as “chromosome shattering” is induced from rupture of micronuclei, followed by fragmentation of micronuclear DNA and its massive rearrangements. **b** Protein stoichiometry imbalance caused by changes in the copy number of chromosomes, leading to proteotoxic stress. **c** Other cellular functions altered by CIN, including metabolic alteration, cell cycle arrest, and senescence

**Fig. 7:**
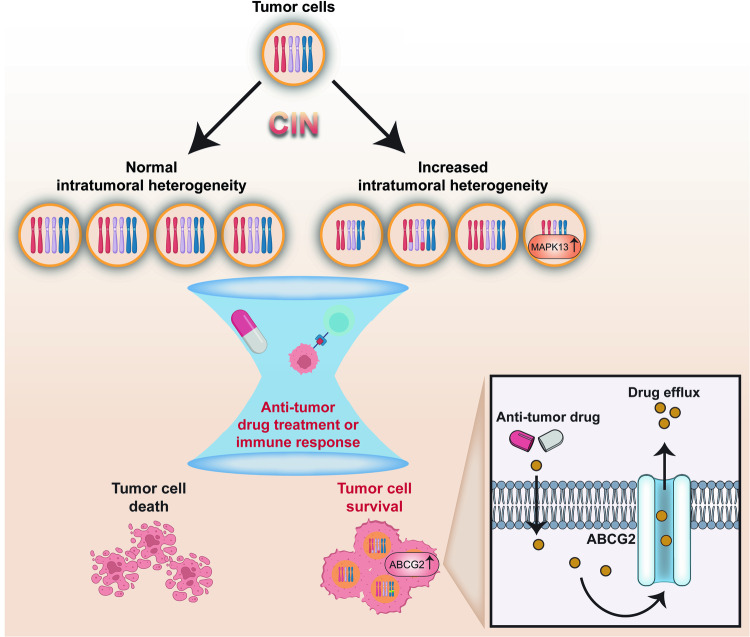
Impact of CIN on drug resistance. CIN confer resistance to anti-tumor drug treatment or immune response through increased intratumor heterogeneity

CIN exerts a multifaceted influence on tumor biology, playing significant roles in both tumorigenesis and tumor progression. However, the relationship between CIN and tumorigenesis is complex and resulting in CIN paradox, as mentioned in the previous section. In this section, we will discuss about the diverse consequences of CIN, starting with those closely related to tumorigenesis. These include DNA damage, proteotoxic stress, and metabolic alteration, which potentially have both beneficial and deleterious effects. We will then explore the generally deleterious effects of CIN on cell cycle arrest and senescence. Lastly, we will discuss the beneficial effects of CIN on metastasis, tumor immune regulation, and drug resistance (Fig. 6, Fig. 7, and Table 2).

**Table 2:** Consequences of CIN and their impact on tumor biology

CIN indicators	Consequence of CIN	Impact on tumor biology	Ref
Lagging chromosome	Formation of micronucleus, aneuploid, or polyploid cells	DNA damage, chromothripsis, proteotoxic stress, metabolic alteration	^70,78,115,329^
Chromosome bridge	Formation of micronucleus, aneuploid, or polyploid cells	DNA damage, chromothripsis, proteotoxic stress, metabolic alteration	^78,115,329^
Micronucleus	DNA damage, chromothripsis	Increased heterogeneity, induction of metastasis through cGAS-STING pathway, tumor immune regulation	^78,115,122–133^
Aneuploidy	Changes in chromosome number or structure	Cell cycle arrest, senescence, metastasis, tumor immune regulation, drug resistance	^271,333–336,361^
Polyploidy	Changes in chromosome number	Cell cycle arrest, senescence, metastasis, tumor immune regulation, drug resistance	^230,252,271,342^

#### DNA damage

CIN can lead to errors in chromosome segregation during cell division, resulting in the formation of lagging chromosomes and chromosome bridges. These lagging chromosome and chromosomal fragments from chromosome bridges often partition into micronuclei, whose nuclear envelope lacks several non-core membrane proteins such as lamin B, lamin B receptor (LBR), and nucleoporins, making them relatively fragile and prone to rupture.^116,129,284–286^ This rupture exposes the DNA content within the micronuclei to the cytoplasm, leading to chromothripsis, also known as “chromosome shattering”, a process of fragmentation of micronuclear chromosome followed by their massive rearrangements into the main nucleus. Hence, while itself a consequence of CIN as it originates from the micronuclear chromosome, chromothripsis could, at the same time, induce the succeeding CIN, as it could lead to the formation of a karyotype with complex chromosomal rearrangements.^78,115,122–133^

Chromothripsis has been associated with various tumor types and can lead to both numerical and structural CIN, altering gene expression patterns and driving changes in cellular behavior.^122,287–289^ Chromothripsis can contribute to tumorigenesis in multiple ways, either beneficial or deleterious. For instance, while DNA damage can be a cause of CIN as discussed in DSBs from replication stress, it is also a significant consequence of CIN, as chromothripsis could induce extensive DNA damage and destabilize tumor cell growth, leading to apoptosis.^122^ Interestingly, it can also create a tumor-promoting environment under certain circumstances, including rearrangement that results in karyotype with a better survival advantage. For example, reparation of shattered chromosomal fragments can lead to the incorporation of genetic material from the shattered fragments into double minute chromosomes, which are small, circular chromosomes without centrosomes distributed asymmetrically to daughter cells during cell division.^290^ Double minutes (also known as extrachromosomal DNA) were reported to contain high copy numbers of *MYC proto-oncogene* (*MYC*). Previously, Martins et al. reported that high MYC is an early event selected in many tumors with CIN, thereby providing a selective growth advantage to the tumor cells.^291–293^ Incorrect repair can also lead to a complete loss of gene function, such as the loss of key tumor suppressor gene *mothers against decapentaplegic homolog 4* (*SMAD4*),^294^ as well as generation of novel oncogenic proteins by chromosome fusion. For example, a novel fusion oncoprotein which could promote AKT signaling activity, *ubiquitin specific peptidase 9 X-linked-ES cell expressed Ras* (*USP9X-ERAS*), is formed by chromothripsis involving the *US9PX* and *ERAS* genes in colon cancer cells.^295^

Collectively, chromothripsis is the consequence and at the same time, the cause of further CIN. The cycle of CIN leading to DNA damage, which in turn exacerbates CIN, forms a complex interaction that plays a crucial role in tumor development and progression. Moreover, recent studies suggest that CIN is associated with changes in both chromatin accessibility and transcription resulting from micronuclei formation.^296,297^ This complex, bidirectional relationship between chromothripsis and CIN, underscores the intricate dynamics of genomic instability in cancer.

#### Proteotoxic stress

Proteins in cells are often composed of more than one subunit. Multi-subunit protein complexes require balanced stoichiometry to function properly.^268,298–303^ This is achieved by regulating the ratio of protein subunits, and by degrading excessive, unassembled protein subunits.^300,304^ Unassembled protein subunits must be bound by chaperones to remain in solution until they are degraded by the ubiquitin-proteasome degradation pathway, thus, excessive production of subunits can overwhelm the protein quality control systems, impairing the stoichiometry and homeostasis of proteins in multi-subunit complexes.^300,304^ Furthermore, the increased demand for degradation puts the cell under proteotoxic stress, a form of cellular stress, and impairs cellular proliferation.^268^ This is often observed in aneuploid cells, where excessive protein subunits encoded by the altered chromosome could lead to imbalanced protein stoichiometry.^305–308^

In contrast, polyploid cells, which contain multiple sets of chromosomes, are likely to suffer less from the effects of genetic alteration and stoichiometry imbalance.^230^ When an essential gene is altered in polyploid cells, they still have more copies of functional genes compared to aneuploid cells. This redundancy provides a buffer against genetic alterations that might otherwise be detrimental. Kuznetsova et al. revealed that tetraploid cells proliferate almost as efficiently as diploid cells, and exhibit only some detrimental phenotypes observed in aneuploid cells.^230^ One reason for this could be that the multiple sets of chromosomes in polyploid cells help maintain a balance in protein stoichiometry by keeping the ratio of subunits forming a protein complex constant;^230^ while in aneuploid cells, gain or loss of certain chromosomes disrupts this ratio, eventually causes the dysfunction of protein complexes that require a specific stoichiometry of their subunits.

Imbalance protein stoichiometry could impair specific cellular functions associated with the affected protein complexes. For example, the gain of chromosome 6, which carries the gene encoding *β-tubulin*, causes lethality in yeast due to excessive β-tubulin production. However, this lethality can be rescued by additional gain of chromosome 13, which carries the *α-tubulin* gene, thus restoring the stoichiometry of α/β-tubulin dimers.^309^ Furthermore, imbalanced protein stoichiometry due to aneuploidy can also lead to the misfolding of proteins, which can accumulate and form toxic aggregates.^268,298–301,310,311^

Paradoxically, aneuploidy could also be beneficial for tumor cells. Aneuploidy could alter protein stoichiometry at the level of the interactome, which is the complete set of molecular interactions within a cell or organism including protein-protein, protein-DNA, and other types of molecular interactions essential for cellular function.^312^ A study of aneuploid patient tumor samples indicated that *MET proto-oncogene, receptor tyrosine kinase* (*MET*) amplification conferred resistance to epidermal growth factor receptor (EGFR) inhibitors, erlotinib and gefitinib.^313^ Typically, MET does not directly activate kinases downstream of the EGFR due to its low binding affinities with them. However, excessive MET expression can bypass the effect of EGFR inhibitors by directly interacting with these kinases and activating them, thereby counteracting the anti-tumorigenic effect of EGFR inhibitors.^313^ This finding illustrates that the aberrant protein interactions, which arise from the excessive proteins due to stoichiometry imbalance, could serve as a mechanism through which aneuploidy reshapes the interactome, thereby promoting tumorigenesis in tumor cells.

#### Metabolic alteration

Metabolism, an essential biological process involving a series of chemical reactions that convert food into energy and building blocks for the cells, is controlled by a finely-tuned coordination of complex metabolic networks, which depends on the precise balance of enzymes and regulators. Introduction of additional gene copies could disrupt this delicate balance, leading to metabolic alterations. Aneuploid cells, whether in yeast, animal models, or human cells, exhibit altered cellular metabolism.^265,299,314,315^ For instance, amplification of chromosome 4 in yeast results in the increase of amino acid levels, except the levels of aspartate and isoleucine, and various tricarboxylic acid cycle intermediates, leading to defects in cell growth.^316^ In MEFs, trisomy in either chromosome 1, 3, 16, or 19 leads to alterations in glutamine metabolism, and subsequently proliferation defects.^265^ Similarly, extra copy of chromosome 3 or 5 can impair human cell proliferation through downregulation in proteins involved in carbohydrate metabolism^264^ Therefore, CIN can lead to alterations in cellular metabolism, which can have detrimental effects on cells.

However, metabolic alteration, also known as metabolic reprogramming, is a hallmark of cancer that can provide several advantages for tumor cells.^317–319^ Metabolic alteration in tumor cells was first observed by Otto Warburg, who noted alterations in the glucose metabolism of tumor cells.^320^ Since then, alterations in other metabolic pathways, such as amino acids and lipid, along with their importance in tumor biology, have also been found.^321,322^ Normal cells produce energy from glucose effectively in the presence of oxygen by coupling glycolysis with oxidative phosphorylation.^320^ In contrast, one of the most well-known tumor cells metabolic reprogramming is the Warburg effect, a phenomenon where tumor cells increase their glucose uptake and glycolysis rate, and prefer glycolysis followed by fermentation, or aerobic glycolysis, instead of glycolysis followed by oxidative phosphorylation even when oxygen is sufficient.^320,323^ This allows tumor cells to cope with the fluctuating oxygen levels often found within tumor tissues.^324^ Moreover, aerobic glycolysis could also meet the increased demands of rapid cell proliferation for essential building blocks such as nucleotides, amino acids, and lipids.^320,325–327^ Furthermore, it could fulfill the demand of highly proliferating tumor cells for cellular reductants such as nicotinamide adenine dinucleotide phosphate (NADPH), which are crucial for lipid biosynthesis, drug resistance, and for scavenging reactive oxygen species (ROS) generated by high proliferation.^320,323,328^

CIN can induce the Warburg effect, triggering metabolic changes that promote tumorigenesis. This is evidenced by a correlation between karyotypic heterogeneity, which serves as an indicator of CIN, and increased consumption of glucose and glutamine, as well as increased production of lactate and glutamate.^22,299,329,330^ However, the association between other metabolic alterations, such as amino acids metabolic alteration and lipid metabolic alteration, and CIN, except the glutamine and glutamate metabolisms,^329^ has not been reported and still needs further investigation. In addition to the alterations in nutrient-related metabolism, CIN also impacts the cellular redox state. Cells with CIN exhibit changes in mitochondrial numbers and activity, typically resulting in increased ROS.^331^ While high levels of ROS can lead to oxidative stress and potential cell death, moderate levels of ROS can promote metastasis.^332^ Therefore, while CIN initially seems detrimental, it can actually benefit tumor cells by providing them with metabolic advantages. This highlights the complex interplay between CIN, metabolic reprogramming, and tumorigenesis.

#### Cell cycle arrest

CIN has been reported to cause cell cycle arrest.^333–336^ Live-cell imaging of human cells with chromosome missegregation demonstrated that missegregation induces cell cycle arrest in a p53-dependent manner.^333–335,337^ Known as the “guardian of the genome”, p53 plays crucial role in controlling cell cycle progression.^338–341^ The tumor suppressive function of p53 is closely related with response to CIN and is critical for determining the fate of cells experiencing CIN.^333–335,342–346^

p53 could suppress the propagation of structural CIN following chromosomal missegregation by inducing cell cycle arrest and apoptosis, thereby limiting the proliferation of aneuploid cells.^270,334,347,348^ p53 inactivation in tumor cells with CIN results in defects in inducing cell cycle arrest and apoptosis, and eventually, in increased tumor heterogeneity.^104,349–351^ This tendency is consistent with the findings in clinical non-small cell lung tumors, whereas *p53*-mutant tumors display more complex karyotypes than their wild-type counterparts.^352,353^ Furthermore, apoptosis observed in CIN mice model, presumably triggered by increased CIN, was rescued upon depletion of p53.^348,354^ In line with these, in a CIN model using SAC-deficient mice, reducing p53 level leads to increased aneuploidy and T-cell lymphoma proliferation, and at the same time, decreased survival.^104,348,349^ The RAS pathway, a critical signaling pathway in cells, is involved in cellular signal transduction, leading to cell growth and division. Overexpression of *Harvey rat sarcoma virus* (*H-RAS*) can induce CIN. However, the activation of RAS and the subsequent induction of CIN can be halted by the activation of p53, resulting in reduced transforming potential in mice model.^355^ This study further highlights the importance of p53 in monitoring and preventing CIN propagation to progenies.

In addition to p53, the stress kinase p38, which is a proline-directed serine/threonine kinases of the MAPK family, also plays a role in controlling the proliferation of aneuploid cells. p38 is activated in response to various stress stimuli, including CIN induction, and can induce cell cycle arrest by several mechanisms, including the upregulation of CDK inhibitors, growth arrest and DNA damage inducible alpha (GADD45α), and cyclin D, as well as the downregulation of CDC25.^333,356–358^ Moreover, p38 can work side-by-side with p53 to limit the progression of cells with CIN. Upon missegregation events, p38 increases the degradation of MDM2, a negative regulator of p53, through phosphorylation. This leads to the stabilization of p53 protein and apoptosis induction, thereby preventing the proliferation of cells with CIN.^333,359^ In addition to chromosomal-related events, the p38/p53 axis can also respond to metabolic stress induced by ROS formation as a consequence of CIN.^333,334,358,360^ These findings highlight the complex interplay between stress pathways, CIN, and cellular responses, underscoring the crucial roles of p53 and p38 in limiting the progression of cells with CIN.

#### Senescence

Cells with DNA damage and chromosome missegregation often became senescent and acquired the senescence-associated secretory phenotype (SASP). CIN enhanced by the treatment with SAC inhibitor could result in senescence, as indicated by the increase of senescence markers, such as p53, p21, p16, and senescence-associated β-galactosidase activity.^271,361–364^ The DDR pathway, activated in response to CIN-induced DNA damage plays a crucial role in this process.^139^ DDR can lead to the upregulation of p53, which in turn activates the expression of p21.^137,365,366^ This subsequently induces cell cycle arrest as a temporary response to allow DNA repair, or senescence as a permanent state of cell cycle arrest when the DNA damage is too severe to be repaired.^367^ Furthermore, CIN could also enhance the level of another senescence marker, p16, with a mechanism which is still unclear.

The consequence of CIN-induced senescent is complex. From a cell-autonomous perspective, senescence is a mechanism of tumor suppression in which aneuploid cells that undergo senescence will stop dividing and unlikely to undergo cellular transformation.^362,363^ In addition, senescent cells could be further cleared through autophagic cell death.^368^ However, CIN-induced senescence also triggers SASP-like gene expression signature, which might contribute to tumorigenesis.^270,369^ The unique secretome from SASP contains biologically active factors that are released into the microenvironment.^370,371^ These factors, including chemokines, cytokines, growth factors, and immune regulators, can induce a positive feedback loop and cause chronic inflammation, which can have dual effects on tumorigenesis.^270^ On one hand, it can act as a defense mechanism against tumors by promoting an anti-tumor immune response. On the other hand, it can contribute to tumorigenesis, as the secretome could activate key transcription factors involved in tumorigenesis, such as RAS.^372^ Furthermore, CIN-induced senescent cells can also increase the migration and invasion capacities of the neighboring tumor cells through SASP, thereby contributing to tumor progression.^369^ Therefore, the effect of CIN-induced senescence and its SASP-like gene expression is complex, resulting in both tumor-suppressive and pro-tumorigenic outcomes.

#### Metastatic capacity

As supported by pre-clinical and clinical data, changes in chromosome copy number could also influence cell motility, matrix degradation, epithelial-mesenchymal transition (EMT), and other processes necessary for metastatic behavior.^373–376^ For instance, tumor cells harboring an extra copy of chromosome 5 displayed increased metastatic capacity, as it leads to the silencing of epithelial cell-adhesion genes and thereby activates EMT.^377^ Meanwhile, the loss of chromosome 16q has been associated with downregulation of E-cadherin (CDH1).^378^ Interestingly, Gao et al. demonstrated that CIN is also associated with mesenchymal-epithelial-transition (MET), a reverse version of EMT required for extravasation and colonization in different tissues in the process of distant metastasis.^378,379^ Loss of chromosome 10p results in the loss of the zinc finger E-box-binding homeobox 1 (*ZEB1*) gene, thus promoting MET, and subsequently metastasis^378^ Furthermore, CIN could drive metastasis by activating Janus kinase/signal transducer and activator of transcription (JAK/STAT) signaling pathway, and through the establishment of local immunosuppressive microenvironment.^380,381^ Moreover, CIN can also indirectly induce EMT. For instance, the loss of chromosome 8p results in the downregulation of 8p-localized genes, such as *N-acylsphingosine amidohydrolase 1* (*ASAH1*), *farnesyl-diphosphate farnesyltransferase 1* (*FDFT1*), *leptin receptor overlapping transcript-like 1* (*LEPROTL1*), *epoxide hydrolase 2* (*EPHX2*), and *BCL2/adenovirus E1B 19* *kDa protein-interacting protein 3-like* (*BNIP3L*), thereby altering the mevalonate and fatty acid metabolic pathways. Disruption of these lipid metabolic pathways in turn increases the activities of small GTPases, such as Ras homolog family member (RHO), Ras-related C3 botulinum toxin substrate (RAC), and rat sarcoma (RAS), and subsequently promotes invasion and metastasis.^378,382,383^ Meanwhile, in the clinical setting, longitudinal studies such as TRACERx, which track the progression of cancer from primary disease to metastasis and recurrence, have reported that elevated CIN correlates with an increase in metastasis and worse survival outcomes, and subsequently poor prognosis.^384–389^ These results demonstrate the positive correlation between CIN and metastasis, as well as poor survival and outcomes. However, it should be noted that a recent study has also reported the anti-metastasis function CIN. For example, changes in chromosome copy number, for example gaining extra copy of chromosome 13 or chromosome 18, could suppress metastasis.^377^ The underlying mechanisms of how these specific chromosomal changes suppress metastasis remain unclear, highlighting the complex role of CIN in tumor metastatic capacity.

#### Tumor immune regulation

CIN has dual activity in immune response, as it is capable of inducing either anti-tumor or pro-tumor immune response. In xenograft models, tumor cells with increased aneuploidy and polyploidy tend to form tumors in immunocompromised mice. However, these tumors either fail to grow or grow more slowly in immunocompetent mice, suggesting the anti-tumor role of CIN-induced immune response.^390^ This could be attributed to genomic alterations that produce neoantigens, which are recognized by the immune cells, and thus activate the adaptive immune response.^391^ Consequently, CIN can activate anti-tumor immune responses, thereby subjecting tumor cells to the selection pressure imposed by the immune system, which subsequently eliminates them.^391^

However, CIN could also contribute to pro-tumor immune response. As a contributor to genomic instability, CIN increases intratumor heterogeneity,^37^ allowing the generation of different tumor cells with variations in antigen presentation, thereby reducing their visibility to the adaptive immune system, which subsequently leads to immune evasion.^30^ Furthermore, micronucleus, as a product of missegregation of chromosomes, could also trigger CIN-related immune regulation.^53,392–396^ Exposure of chromosomal double-stranded DNA (dsDNA) in the micronuclei to the cytoplasm activates the cyclic GMP-AMP synthase-stimulator of interferon genes (cGAS-STING)-dependent immune response, a type of innate immune response originally discovered as a sensor of viral dsDNA.^53,397–400^ Typically, recognition of dsDNA by cGAS activates STING, which could in turn activate anti-tumor immune response through type 1 interferon (IFN) and canonical nuclear factor kappa B (NF-κB) signaling.^53,392,401,402^ Interestingly, in the context of CIN, STING promotes EMT and the expression of inflammatory genes, enhancing cell migratory capacity, and subsequently promoting metastasis, by activating non-canonical NF-κB signaling.^53,392,403^ This process indicates that CIN manipulates the innate immune system to promote tumor cells immune evasion.^53^ Furthermore, this example clearly demonstrates that CIN could alter normal cell function to favor tumor progression. Moreover, the activation of SASP through CIN-induced senescence could also shift the immune response towards pro-tumor.^372^ Together, these observations suggest that CIN, through increased heterogeneity or by activation of pathways in favor of pro-tumor immune response, can promote tumor cells immune evasion.

#### Drug resistance

Anti-tumor drugs encompass a wide range, each with its own complex mechanisms. For instance, alkylating agents cause DNA crosslinks that disrupt DNA replication, eventually inhibiting tumor cell division,^13,317,401,402^ while anti-metabolites can cause improper DNA synthesis by mimicking endogenous molecules.^19,127^ Irrespective of their mechanism, resistance to anti-tumor drugs has become a significant hurdle in anti-tumor treatment.^27,404^ While the molecular mechanism of tumor cells drug resistance are complex and have not been totally elucidated, CIN, as a fuel of tumor evolution that causes intratumor heterogeneity, has been assumed as one of the major reason for drug resistance.^230,336,405,406^ This increase in heterogeneity can be viewed as a survival strategy employed by tumor cells to adapt to unpredictable environments. This strategy, known as biological bet-hedging or “not putting all your eggs in one basket”, allows tumor cells to diversify their phenotypes, spreading the risk and increasing the likelihood of some cells to survive under selective pressures, such as those imposed by anti-tumor drug treatments.^23,407,408^

Moreover, Ippolito et al. previously reported the link between CIN and drug resistance through the upregulation of *ATP binding cassette subfamily G member 2* (*ABCG2*), a drug efflux pump, due to the amplification of its upstream regulator *MAPK13* in topotecan-resistant tumor cells generated from treatment using CIN-inducing drug nocodazole.^17^ Together, these show that CIN fuels genomic diversity, upon which selection works, leading to the development of drug resistance.

Although the result from the aforementioned study suggests that the generation of intratumor heterogeneity through CIN can shield tumor cells from the selective pressure caused by anti-tumor drugs, the role of CIN in drug response is nevertheless a complex relationship, as CIN could potentially be induced to excessive level, leading to cell death and enhancing the effectiveness of anti-tumor drug treatments. The following section will discuss how the induction of CIN can be leveraged to enhance the efficacy of anti-tumor drugs. A summary of the different types of anti-tumor drugs and their interactions with CIN is provided in Table 3.

**Table 3:** Anti-tumor drugs and CIN-related drug resistance mechanism

Class	Mechanism of Action	Examples	CIN-modulated resistance mechanism	Ref
Anti-metabolites	Mimic endogenous molecules, causes improper DNA synthesis	Methotrexate, cladribine, fluorouracil, cytarabine, mercaptopurine, fludarabine	Increased heterogeneity	^19,127^
Alkylating agents	Form DNA strand cross-links, induce DNA damage	Myleran, chlorambucil, cisplatin, oxaliplatin	Increased DNA repair mechanisms, delayed cell cycle	^13,317,401,402^
Topoisomerase inhibitors	Inhibits DNA unwinding during replication or transcription	Irinotecan, topotecan, etoposide, teniposide	Upregulation of drug efflux pumps	^17,406^
Mitotic inhibitors	Inhibits tubulin polymerization	Vincristine, vinblastine, docetaxel, paclitaxel	Alterations in SAC, delayed cell cycle	^336^
Anthracyclines	DNA intercalation, induce DNA damage	Doxorubicin, idarubicin, daunorubicin, epirubicin,	Increased DNA repair mechanisms	^18,230,405,406^
Protein kinase inhibitors	Blocks the action of protein kinases	Imatinib, dasatinib, nilotinib, gefitinib, vemurafenib, trametinib	Alterations in signaling pathways	^18,272,478,479^
Proteasome inhibitors	Induces ER stress due to accumulation of misfolded proteins	Bortezomib, carfilzomib, ixazomib	Upregulation of drug efflux pumps	^480–482^
PARP inhibitors	Block PARP-dependent DNA repair mechanism	Olaparib, rucaparib	Alterations in DNA repair mechanisms	^406^
Monoclonal antibodies	Blocks specific signaling pathway, trigger anti-tumor immune response	Rituximab, trastuzumab, cetuximab, nivolumab, pembrolizumab	Alterations in antigen expression	^483^

### CIN-based potential anti-tumor therapy

Previous studies indicated that an excessive level of CIN beyond a certain threshold could potentially induce tumor cell death. Thus, enhancing the CIN level has been proposed as a promising approach to target tumor cells, and strategies to exacerbate CIN for anti-tumor therapy have been explored.^66,358,406,409–422^ For example, taxol could increase the number and severity of chromosome segregation errors in tumor cells, while cells with excessive CIN were more sensitive to low doses of taxol.^423^ Indeed, combining taxol and monopolar spindle 1 (MPS1) inhibitor could reduce xenograft growth more effectively than either compound alone.^411^ Furthermore, combining SAC inhibitor and other non-taxol-based compounds that induce CIN can synergistically reduce tumor growth. For example, combining a SAC inhibitor with microtubule-destabilizing drug SKI606 results in the selective killing of tumor cells exhibiting a CIN phenotype.^410^ Moreover, combining a p38α inhibitor, which interferes with DNA damage response, with taxane-based chemotherapy increased the efficiency of killing breast tumor cells compared to taxanes alone by boosting CIN.^358^ While they have not yet been used in clinical settings, there are also other compounds that can induce CIN. These include inhibitors of the SAC proteins MAD2 or BUBR1, which can induce tumor cell death. Moreover, a compound that induces CIN by targeting the highly expressed in cancer 1/NIMA-related kinase 2 (Hec1/Nek2)-related mitotic pathway also demonstrates promising results.^424–426^

While enhancing CIN could potentially be a powerful method to eradicate tumors, the feasibility of such therapies will depend on many factors including CIN status and the capacity of the tumor cells to tolerate CIN.^267,427–437^ Moreover, it is important to consider that untransformed cells will also be affected by the CIN-inducing agents and thus will suffer from low to moderate CIN rates. This may predispose these cells to become tumorigenic, leading to therapy-induced tumorigenesis subsequently.

To overcome this problem, efforts have been made to develop strategies that more selectively target cells displaying CIN phenotype, either by exploiting specific vulnerabilities of tumor cells with CIN or identifying new weaknesses incurred in tumor cells with new karyotype. For example, a study by Marquis et al., which aims to identify synthetic lethal gene in tumor cells with CIN, has discovered that targeting kinesin family member 18A (KIF18A) is particularly detrimental to aneuploid tumor cells.^438^ This sensitivity arises from alterations in spindle geometry and microtubule dynamics specific in tumor cell with CIN, which, upon *KIF18A* knockdown, leads to excessive CIN and reduces tumor cells viability. This suggests that KIF18A could be a promising synthetic lethal candidate for future drug development efforts targeting tumor cells with CIN. Furthermore, Hong et al. found that IL-6/STAT3 signaling axis downstream of cGAS-STING enables the survival of tumor cells with CIN, and blockade of IL-6 signaling by tocilizumab, a clinically used drug that targets the IL-6 receptor (IL-6R), can impair their growth specifically.^403^ Moreover, aneuploid cells have also been found to contain higher levels of ceramide, and further increasing the levels of ceramide through treatment with N-[2-hydroxy-1-(4-morpholinylmethyl)-2-phenylethyl]-decanamide monohydrochloride (DL-PDMP), an antagonist of UDP-glucose ceramide glucosyltransferase, is significantly more toxic to aneuploid cells compared with diploid cells.^439^

Another strategy utilizes new weakness that results from gaining specific chromosome. For instance, a gene encoded on chromosome 1, known as *uridine-cytidine kinase 2* (*UCK2*), is required to activate certain pro-drugs, such as RX-3117 and 3-deazauridine. A recent study revealed that cells with an extra copy of chromosome 1 express higher level of UCK2 and are more sensitive to those drugs compared to diploid cells with just two copies,^440^ suggesting that introducing specific aneuploidies that can exert anti-tumor function, for example using CRISPR-based tools, might also be a potential CIN-based anti-tumor therapeutic strategy.^441–447^ This strategy is still not yet translated to clinical trial, nevertheless, several compounds targeting different pathways to increase CIN are now in phase I-III clinical trials,^448–450^ representing promising progress for future research and development in CIN-based anti-tumor therapy (Table 4).

**Table 4:** Clinical trials of CIN-inducing drugs for anti-tumor treatment

Inhibitors	Drug type	Effect on CIN	Identifier/status/phase	Ref
MLN8237 (Alisertib)	AURKA inhibitor	Abnormal spindle poles	NCT00500903 completed I	^484,485^
			NCT00853307 completed II	^486^
			NCT01653028 completed II	^487^
			NCT01799278 completed II	^488^
			NCT01091428 completed I/II	^489^
			NCT02038647 completed II	^490^
			NCT02109328 completed II	^491^
			NCT01094288 completed I	^492^
			NCT01639911 completed I	^493^
			NCT01601535 completed I	^494^
			NCT02319018 completed II	^495^
			NCT02219789 completed I	^496^
			NCT02293005 ongoing I/II	^497,498^
			NCT01924260 completed I	^499^
			NCT02187991 completed I	^500^
			NCT02719691 completed I	^501^
			NCT04555837 completed I	^502^
			NCT04479306 completed I	^503,504^
			NCT04085315 completedI	^505^
			NCT00697346 completed I	^506^
			NCT01466881 completed II	^507^
			NCT01482962 completed III	^508^
			NCT00807495 completed II	^509^
			NCT01034553 completed I/II	^510,511^
			NCT02560025 completed II	^512^
			NCT01695941 completed I	^510^
			NCT02444884 completed I	^513^
			NCT01154816 completed II	^514^
			NCT02114229 completed II	^515,516^
ENMD-2076	AURKA inhibitor		NCT00658671 completed I	^517,518^
			NCT01104675 completed I	^519^
			NCT01914510 completed I	^520^
			NCT01719744 completed II	^521^
			NCT01639248 completed II	^522^
			NCT02234986 completed II	^523^
			NCT00904787 completed I	^524^
LY3295668	AURKA inhibitor		NCT03092934 completed I	^525^
			NCT03955939 completed I/II	^526^
			NCT03898791 completed I	^527^
			NCT04106219 completed I	^528^
MLN8054	AURKA inhibitor		NCT00249301 completed I	^529^
			NCT00652158 completed I	^530^
MK-5108	AURKA inhibitor		NCT00543387 completed I	^531,532^
TAS-119	AURKA inhibitor		NCT02448589 completed I	^533,534^
			NCT02134067 completed I	^535^
KW-2449	AURKA inhibitor		NCT00346632 completed I	^536^
			NCT00779480 completed I	^497,537^
AZD1152 (Barasertib)	AURKB inhibitor	Abnormal spindle poles	NCT00338182 completed I	^538,539^
			NCT00497731 completed I	^540^
			NCT00497991 completed I/II	^541^
			NCT00530699 completed I	^542^
			NCT00952588 completed I	^543^
			NCT01019161 completed I	^544^
			NCT00926731 completed I	^545^
			NCT03217838 completed I/II	^546^
			NCT01354392 completed II	^546^
BI-831266	AURKB inhibitor		NCT00756223 completed I	^547^
BI-811283	AURKB inhibitor		NCT00701324 completed I	^548^
			NCT00632749 completed II	^549^
Chiauranib	AURKB inhibitor		NCT02122809 completed I	^550,551^
			NCT03901118 ongoing II	^497^
			NCT03245190 ongoing I/II	^552^
			NCT03974243 ongoing I/II	^553^
			NCT03216343 completed I/II	^554^
AT9283	AURKA/B inhibitor	Abnormal spindle poles	NCT00443976 completed I	^555,556^
			NCT00522990 completed II	^557^
			NCT00985868 completed I	^558^
Cenisertib	AURKA/B inhibitor		NCT00391521 completed I	^559,560^
			NCT01097512 completed I	^561^
			NCT01080664 completed I	^562^
CYC116	AURKA/B inhibitor		NCT00560716 completed I	^563^
PF-03814735	AURKA/B inhibitor		NCT00424632 completed I	^564,565^
TAK-901	AURKA/B inhibitor		NCT00935844 completed I	^566^
			NCT00807677 completed I	^566^
Danusertib	AURKA/B/C inhibitor	Abnormal spindle poles	NCT00872300 completed II	^567^
			NCT00766324 completed II	^568^
GSK1070916 (NIM-900)	AURKB/C inhibitor		NCT01118611 ongoing I	^569^
ABT-348 (Ilorasertib)	AURKA/B/C inhibitor		NCT01110486 completed I	^570,571^
			NCT01110473 completed I	^572^
			NCT02478320 completed II	^571^
AMG-900	AURKA/B/C inhibitor		NCT00858377 completed I	^573,574^
			NCT01380756 completed I	^575^
BI-847325	AURKA/B/C inhibitor		NCT01324830 completed I	^576,577^
SNS-314 Mesylate	AURKA/B/C inhibitor		NCT00519662 completed I	^578^
Tozasertib	AURKA/B/C inhibitor		NCT00500006 completed I	^579^
			NCT02532868 completed I	^580^
			NCT00111683 completed I	^581^
GSK923295	CENP-E inhibitor	Metaphase misalignment	NCT00504790 completed I	^582,583^
Ispinesib	KIF11 inhibitor	Monopolar spindle	NCT00169520 completed I/II	^584^
			NCT00119171 completed I/II	^584^
			NCT00136578 completed I/II	^585^
			NCT00095992 completed I	^586^
			NCT00096499 completed I	^587^
			NCT00095628 completed II	^588^
			NCT00095953 completed II	^589^
			NCT00354250 completed II	^590^
			NCT00097409 completed II	^588^
			NCT00607841 completed I	^591^
			NCT00363272 ongoing I	^592^
SB-743921	KIF11 inhibitor		NCT00136513 completed I/II	^593^
			NCT00343564 completed I/II	^594^
Filanesib	KIF11 inhibitor		NCT00462358 completed I	^595^
			NCT00637052 completed I/II	^596^
			NCT00821249 completed I/II	^597^
			NCT01248923 completed I	^598^
			NCT01372540 completed I	^599^
			NCT02384083 completed I/II	^600^
ALN-VSP02	KIF11 inhibitor		NCT01158079 completed I	^601^
			NCT00882180 completed I	^601^
Litronesib	KIF11 inhibitor		NCT01214629 completed I	^602^
			NCT01214642 completed I	^603^
			NCT01358019 completed I	^604^
4SC-205	KIF11 inhibitor		NCT01065025 completed I	^605^
AZD4877	KIF11 inhibitor		NCT00613652 completed I	^606^
			NCT00389389 completed I	^607^
			NCT00486265 completed I	^608^
			NCT00661609 completed II	^609^
ARQ 621	KIF11 inhibitor		NCT00825487 completed I	^585^
MK-0731	KIF11 inhibitor		NCT00104364 completed I	^610^
VLS-1488	KIF18A inhibitor		NCT05902988 ongoing I/II	^68^
CFI-402257	Mps1 inhibitor	Chromosome missegregation	NCT02792465 completed I	^64^
			NCT03568422 completed I	^611^
BAY1161909 (Empesertib)	Mps1 inhibitor		NCT02138812 completed I	^612^
BAY1217389	Mps1 inhibitor		NCT02366949 completed I	^613^
S81694	Mps1 inhibitor		NCT03411161 completed I/II	^614^
BOS172722	Mps1 inhibitor		NCT03328494 completed I	^615,616^
Olaparib	PARP inhibitor	Disrupted DNA repair system	NCT01623349 completed I	^617^
Rucaparib	PARP inhibitor		NCT03654833 completed II	^618^
BI 2536	Plk1 inhibitor	Premature separation of sister chromatids	NCT00376623 completed I	^619,620^
			NCT02211833 completed I	^621^
			NCT00710710 completed II	^622^
			NCT00701766 completed I/II	^623^
			NCT00243087 completed I	^624^
BI6727 (Volastertib)	Plk1 inhibitor		NCT02273388 completed I	^625^
			NCT00969553 completed I	^626^
			NCT01348347 completed I	^627^
			NCT01022853 completed I	^628^
			NCT01206816 completed I	^629^
			NCT00969761 completed I	^630^
			NCT01662505 completed I	^631^
			NCT01023958 completed II	^632^
			NCT01121406 completed II	^632^
			NCT00824408 completed II	^633^
			NCT00804856 completed II	^634^
			NCT01721876 completed III	^635,636^
ON 01910.Na (Rigosertib)	Plk1 inhibitor		NCT01125891 completed I	^637^
			NCT01926587 completed I//II	^638^
			NCT03786237 completed I	^612,639^
			NCT04177498 completed I	^612,639^
			NCT04263090 completed I	^640^
			NCT02562443 ongoing III	^641^
			NCT01360853 completed III	^642^
			NCT01241500 completed III	^641^
			NCT01928537 completed III	^643^
GSK461364	Plk1 inhibitor		NCT00536835 completed I	^644,645^
MK-1496	Plk1 inhibitor		NCT00880568 completed I	^612^
TAK-960	Plk1 inhibitor		NCT01179399 completed I	^646,647^
NMS-1286937 (Onvansertib)	Plk1 inhibitor		NCT01014429 completed I	^648,649^
			NCT03303339 completed I/II	^650^
			NCT03829410 completed I/II	^651^
			NCT03414034 completed I/II	^652,653^
TKM-080301	Plk1 inhibitor		NCT02191878 completed I/II	^654,655^
			NCT01262235 completed I/II	^656^
			NCT01437007 completed I/II	^657,658^
CYC 140	Plk1 inhibitor		NCT03884829 ongoing I	^659^
CFI-400945	Plk4 inhibitor		NCT01954316 completed II	^449,660^
AZD1775	WEE1 inhibitor	Premature entry into mitosis	NCT03253679 completed II	^661,662^
Adavosertib	WEE1 inhibitor		NCT02101775 completed II	^663,664^

## Conclusion

Great progress has been made during this last decade in understanding how chromosome segregation is regulated and what defects might contribute to chromosome missegregation in tumor cells. While the exact source of CIN in tumor cells is complex and diverse, it is clear that a variety of mechanisms can lead to this phenotype. Tumor cells presumably employ different routes to achieve the same CIN phenotype. Therefore, more research to further characterize the mechanisms contributing to CIN is necessary. This will be crucial in laying the foundation for developing strategies that can modulate CIN, with the aim of inhibiting tumor cells ability to adapt to environmental challenges, as well as preventing tumor drug resistance.

From a clinical perspective, although the relative contribution of various mechanisms of CIN has been described in cultured tumor cell lines, whether these models also recapitulate the types of CIN found in primary and metastatic tumors, as well as the epigenetic causes and consequences of CIN, remains to be determined. Enhancing CIN has been proposed as a promising approach to target aneuploid tumor cells. From the perspective of developing new therapeutic strategies, it is crucial to determine whether cells undergoing CIN share common characteristics. It is also important to understand if CIN triggers a common stress response and whether CIN cells need to develop specific adaptations to adapt to their altered genomes. A more comprehensive understanding of these effects and the resulting cellular responses is crucial for the successful exploitation of CIN for therapeutic strategies. Furthermore, while several SAC inhibitors that increase CIN show promising potential for reducing tumor growth and have already entered clinical trial phase, the success of such therapies depends on factors such as CIN status and the capacity of the tumor cells to tolerate CIN.^267,427–437^ Despite these challenges, recent advances suggest promising possibilities for a new, personalized, CIN-specific approach to anti-tumor therapy.^358,406,410,411,423–426,438,451–455^

Moreover, considering CIN as the fuel for genomic diversity, it is important to acknowledge the challenges posed by tumor evolution. This suggests that future research could focus on characterizing the common phenotypes that emerge from various CIN routes. These strategies might not directly target the mechanisms of CIN; instead, they could focus on the phenotypes selected during the stages of tumor initiation and progression. Adopting this approach could potentially lead to more effective treatments that are less susceptible to being undermined by tumor adaptation and evolution. Furthermore, it could provide valuable biomarkers for early detection and prognosis, thereby opening up new opportunities for preventive measures.^293^

Furthermore, while targeting tumor cells with CIN holds promise, it is important to consider the risks. Increasing CIN in untransformed cells could inadvertently lead to therapy-induced tumor due to their potential to become tumorigenic under low to moderate CIN rates. Therefore, future research should aim to selectively target cells with a CIN phenotype, thereby reducing the risk of unintentional transformation of normal cells.

In conclusion, our understanding of CIN and its role in tumorigenesis has greatly improved. Although many questions remain unanswered, and further research is needed to fully understand the mechanisms underlying CIN-related tumorigenesis as well as its potential as a therapeutic target, the study of CIN and its effects on tumor cells have nevertheless laid a promising foundation. These insights could potentially guide the development of new strategies for diagnosing and treating cancer.
